# Matter of TIME: the tumor-immune microenvironment of mesothelioma and implications for checkpoint blockade efficacy

**DOI:** 10.1136/jitc-2021-003032

**Published:** 2021-09-13

**Authors:** James Harber, Tamihiro Kamata, Catrin Pritchard, Dean Fennell

**Affiliations:** Cancer Research Centre, University of Leicester College of Life Sciences, Leicester, UK

**Keywords:** macrophages, CD8-positive T lymphocytes, tumor microenvironment, immunotherapy, lymphocytes, tumor-infiltrating

## Abstract

Malignant pleural mesothelioma (MPM) is an incurable cancer with a dismal prognosis and few effective treatment options. Nonetheless, recent positive phase III trial results for immune checkpoint blockade (ICB) in MPM herald a new dawn in the fight to advance effective treatments for this cancer. Tumor mutation burden (TMB) has been widely reported to predict ICB in other cancers, but MPM is considered a low-TMB tumor. Similarly, tumor programmed death-ligand 1 (PD-L1) expression has not been proven predictive in phase III clinical trials in MPM. Consequently, the precise mechanisms that determine response to immunotherapy in this cancer remain unknown. The present review therefore aimed to synthesize our current understanding of the tumor immune microenvironment in MPM and reflects on how specific cellular features might impact immunotherapy responses or lead to resistance. This approach will inform stratified approaches to therapy and advance immunotherapy combinations in MPM to improve clinical outcomes further.

## Immunity in the causation and therapy of malignant pleural mesothelioma (MPM)

MPM is causally associated with exposure to asbestos fibers.^1^ From its initiation, the immune system is intimately involved in MPM. Mechanistically, carcinogenesis is thought to involve unsuccessful clearance of asbestos fibers by phagocytic macrophages in a process termed ‘frustrated phagocytosis’,^2^ followed by continuous amplification of proinflammatory cytokine and paracrine high mobility group box 1 (HMGB1) signaling between affected mesothelial cells.^3^ Coupled with the biopersistence and phagocytosis resistance of these fibers, these processes result in chronic inflammation, oxygen radical release and DNA damage, leading to malignant transformation. MPM tumors subsequently co-ordinate tumor-associated macrophages (TAMs), myeloid-derived suppressor cells (MDSCs) and regulatory T cells (T_regs_) to achieve an immunosuppressive microenvironment favorable for their survival.

**Figure 1:**
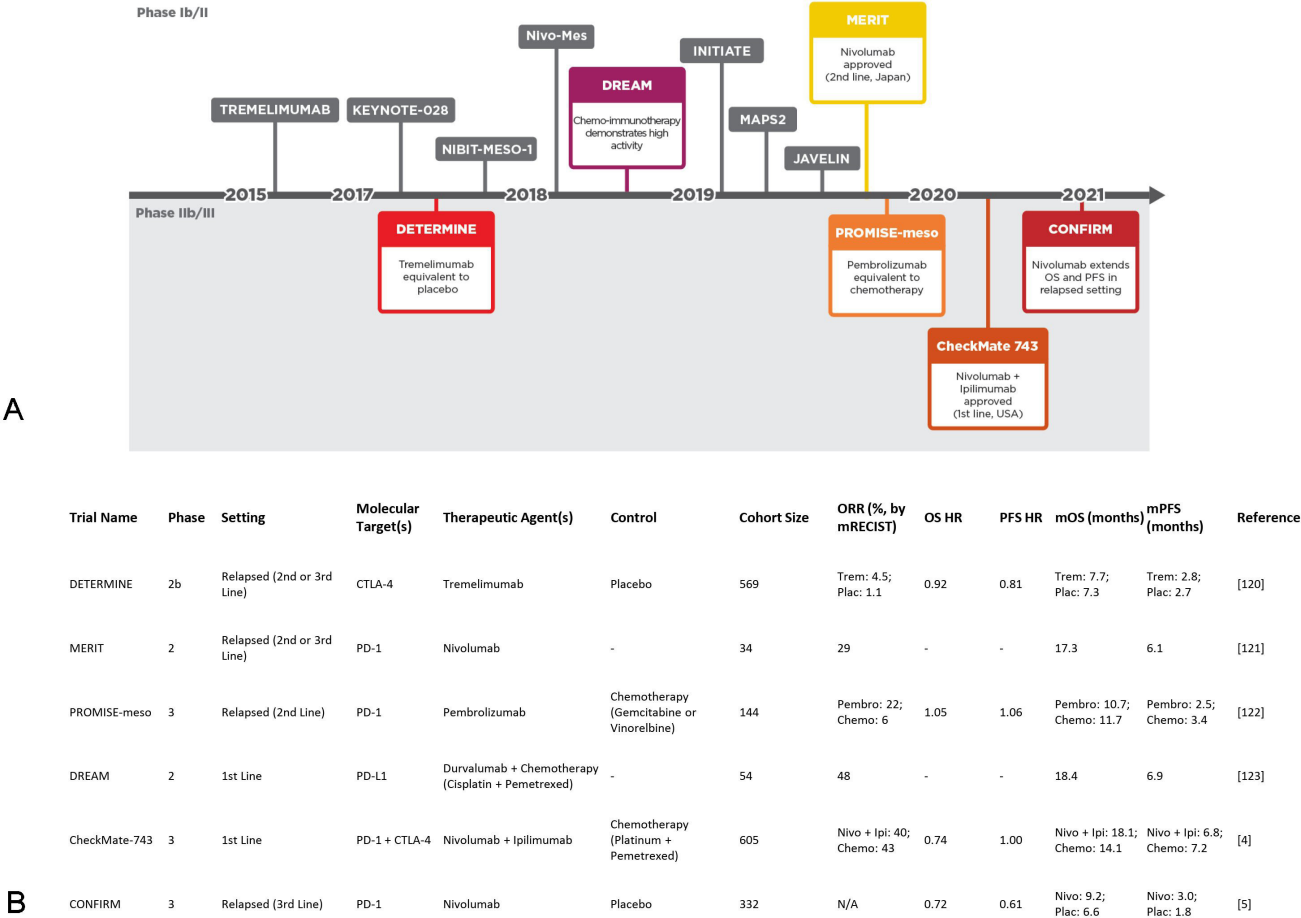
ICB has revolutionized the MPM clinical landscape. (A) Clinical trials of ICB interventions organized on a timeline according to initial publication of results.^4 5 120–123^ (B) Details of ICB trials highlighted in A. Chemo, chemotherapy; ICB, immune checkpoint blockade; Ipi, ipilimumab; mOS, median overall survival; mPFS, median progression-free survival; MPM, malignant pleural mesothelioma; N/A, not available at time of writing; Nivo, nivolumab; ORR, object response rate; Pembro, pembrolizumab; Plac, placebo; Trem, tremelimumab.

Immune checkpoint blockade (ICB) partially lifts immunosuppression by disrupting T-cell inhibitory receptor ligation and has gained regulatory approval in several cancers. More recently, ICB has become the only newly approved systemic treatment for MPM in 16 years (figure 1). The CheckMate 743 trial evaluated nivolumab and ipilimumab (anti-PD-1 and anti-CTLA-4 [cytotoxic T lymphocyte-associated protein 4], respectively) combined ICB and reported significantly improved overall survival (OS) versus standard of care chemotherapy (cisplatin or carboplatin plus pemetrexed) in the first-line setting, particularly in non-epithelioid tumors.^4^ Moreover, the CONFIRM (**C**heckp**o**i**n**t Blockade **f**or **I**nhibition of **R**elapsed **M**esothelioma) trial of nivolumab vs placebo was the first randomized phase III trial to show improved survival in relapsed MPM.^5^ While ICB has already gained regulatory approval, alternative immunotherapeutic approaches such as chimeric antigen receptor T cells and vaccine-based treatments are also showing promise.^6 7^ The broader immunotherapeutic landscape of MPM has been comprehensively reviewed recently.^8–12^

The intimate involvement and abundance of leukocytes in MPM^13^ from carcinogenesis to diagnosis suggest that the tumor immune microenvironment’s (TIME’s) composition may influence response to immunotherapy^14^ and the tumor’s genomic landscape.^15^ This review article will focus on cytotoxic T-lymphocyte (CTL) biology and the specific role of TAMs in immunosuppression. The influence of MDSCs, T_regs_ and MPM-intrinsic modulation of the TIME will also be discussed. Since this is a broad and complex topic, the roles of the TIME’s other components are beyond the scope of this current review but have been described recently.^16–18^

## CTL phenotypes and prognosis in MPM

CTLs (ie, CD8^+^ cells) are tumor-suppressive and play a critical role in the effectiveness of ICB in cancer.^19^ In MPM, CTLs typically represent approximately 5%–15% of the total immune infiltrate,^20 21^ while CTL deserts are rare.^22^ One study reported more CD8^+^ cells in PD-L1^+^ tumors than PD-L1^−^ ones,^23^ implying that this routinely used biomarker may also predict CTL infiltration. Additionally, there is evidence that the ratio of CTLs to malignant cells is higher in sarcomatoid MPM.^24^ Furthermore, by examining paired biopsies, Pasello and coworkers detected that the CD8^+^ cell proportion increased following administration of platinum plus pemetrexed.^25^ Finally, our own group has recently demonstrated via multiregional sequencing that CTL infiltration can be influenced by the tumor’s clonal architecture.^26^

**Figure 2:**
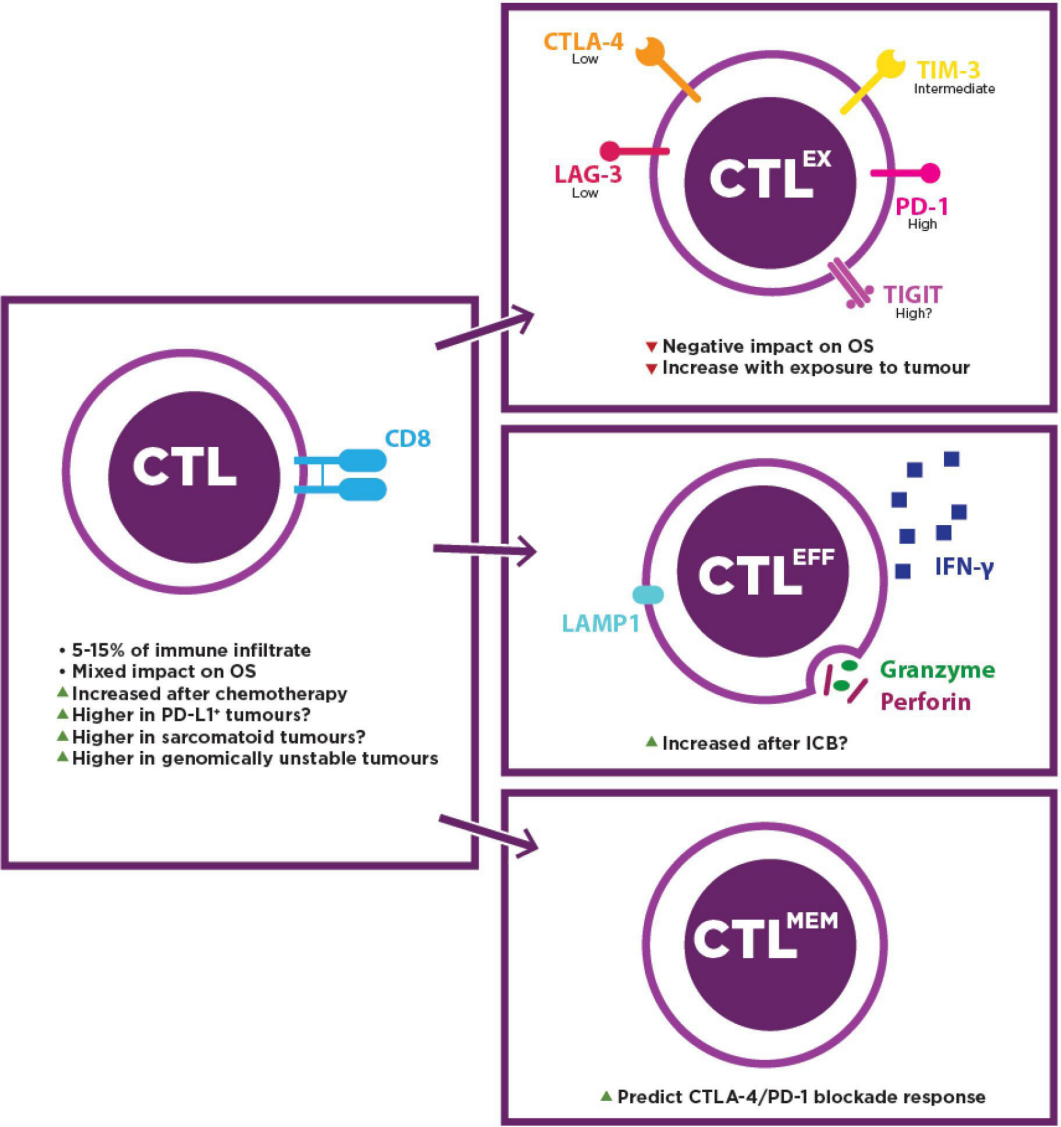
Profile of CTL characteristics and clinical impacts as detected in MPM. Low, intermediate and high (top right panel) indicate relative levels of inhibitory receptors reported thus far in MPM. A ? indicates that the observation is tentative, being based on conflicting reports or requiring corroboration. CTL, cytotoxic T lymphocyte; CTL^EFF^, effector CTL; CTL^MEM^, memory CTL; CTL^EX^, exhausted CTL; ICB, immune checkpoint blockade; 'IFN-γ', interferon gamma; MPM, malignant pleural mesothelioma; OS, overall survival.

Although CTLs are tumoricidal, their quantity alone does not relate to clinical benefit (figure 2). Positive correlation of infiltrating CD8^+^ cells with OS has been reported,^27 28^ yet not uniformly,^29 30^ with one tissue microarray study of advanced, non-epithelioid MPMs even showing poorer OS in the univariate, CD8^+^-high group.^31^ These discordant findings may result from the heterogeneous distribution of CTLs and resulting sampling bias^32^ or, alternatively, from functional variability.

Effector CTLs are primed for cytotoxicity and release high levels of perforin and granzyme, which are required to induce apoptosis in the target cell. Although functional characterisation remains relatively underexplored, current evidence suggests CTLs are responsive despite the unfavorable TIME in MPM. One group showed that peripheral CTLs from patients with MPM exhibit higher perforin expression than healthy volunteers’, though no difference was observed after PMA/ionomycin stimulation.^33^ Elsewhere, Wu *et al* reported greater perforin and granzyme expression after radiation therapy plus CTLA-4 blockade compared with radiation alone in the AB12 biphasic murine model, suggesting that the effector phenotype had arisen due to ICB.^34^ A similar observation was made in a peripheral blood mononuclear cell (PBMC):MPM coculture system: PD-1 axis inhibition with or without TIM-3 blockade stimulated superior cytokine and granzyme B secretion compared with alternative ICB combinations and controls.^35^

Alternatively, CD8^+^ cells can be exhausted CTLs, a languid class of lymphocyte that diverges from effector CTLs due to weak, chronic antigenic stimulation,^36 37^ typically characterized by relatively high expression of multiple inhibitory receptors such as PD-1, CTLA-4, TIM-3, LAG-3 and TIGIT, etc, along with a NFAT2/TOX-driven epigenetic program.^38^ Moreover, they underperform their effector cousins regarding cognate epitope reactivity, cytotoxic activity and cytokine secretion.^37^

In MPM, one study found TIM-3 and TIGIT protein upregulated on CD8^+^ cells when compared with tumor-free lung-associated CTLs, while approximately 60% were PD-1 positive.^20^ PD-1, LAG-3 and TIM-3 protein on CTLs were found to be elevated in MPM samples versus pleuritis,^30^ suggesting that exhaustion may be widespread and a consequence of multiple, redundant interactions in this disease. Indeed, one study discovered increasing expression of multiple inhibitory receptors with elapsed time in the AE17sOVA murine MPM model.^39^ Notably, CTLA-4 expression seems to be lower than other immune checkpoint (ICs) on MPM-associated CTLs,^20 30^ which may partially explain the disappointing results of anti-CTLA-4 monotherapy. Likewise, despite multiple studies identifying LAG-3 in MPM tumors and pleural effusions,^30 40^ it is generally less commonly expressed than other inhibitory receptors, particularly within the tumor,^21 41^ and a detailed examination of CTL immune checkpoints revealed that LAG-3 tended to be a companion to PD-1 rather than a solo exhaustion marker.^42^ Hence, an anti-LAG-3 therapeutic strategy for CTLs may not be favored at present but merits further investigation. Conversely, TIM-3 expression is moderate on CTLs in MPM, and CD8^+^/TIM-3^+^ PBMCs exert a negative effect on OS in patients receiving CTLA-4 inhibitors.^43^ Combined with PD-1 axis inhibition, TIM-3 blockade can induce a tumoricidal effector phenotype in PBMCs cocultured with MPM cells.^35^ In summary, TIM-3 blockade may provide clinical efficacy alongside PD-1 axis inhibition and in tumors refractory to existing therapies. Widespread inhibitory receptor expression could facilitate vigorous antitumor immune responses in patients receiving combination or successive ICB, as demonstrated in melanoma, where high TIM-3 expression has been associated with PD-1 and CTLA-4 blockade resistance.^44^

Memory subset predomination among MPM-infiltrating CTLs has also been suggested.^20^ Immunophenotyping of PBMCs before ICB has suggested that a high CD45RA^+^/CCR7^−^ effector memory CTL subpopulation can predict response to combined PD-1 and CTLA-4 inhibition, along with a low naïve memory subpopulation.^45^ However, validation of their contribution to durable responses, as well as the impact of tumor-infiltrating memory CTLs on therapeutic outcomes in MPM, requires further investigation.

### CTL-derived IFN-γ is attenuated in MPM

On T-cell receptor engagement, effector CTLs secrete IFN-γ, which activates an antitumor phenotype in natural killer cells and macrophages^46^ and also upregulates tumor-surface major histocompatibility complex class I (MHC I) to support neoantigen presentation. A CRISPR (clustered regularly interspaced short palindromic repeats) screen in the Renca mouse model also identified IFN-γ downregulators as key mediators of CTL evasion by tumor cells.^47^ Thus, IFN-γ is a crucial cytokine for effective antitumor immunity.

In MPM, one study found that tumor-infiltrating CTLs produce significantly less IFN-γ when stimulated than both patient-matched circulating ones and those present in tumor-free lung samples.^20^ Conversely, Khanna and colleagues demonstrated that lymphocytes isolated from pleural effusions of patients with MPM (rather than tumor-infiltrating ones) were reactive to autologous tumor cells, exhibiting increased production of IFN-γ when coincubated.^48^ Together, these results suggest that the capacity of CTLs to produce IFN-γ may be attenuated by extended exposure to the MPM tumor or its TIME, as discussed later. In agreement with this, a study in the AE17sOVA MPM model found that IFN-γ production was markedly decreased in CTLs stimulated after isolation at 22 days postinfection compared with those collected after 15 days.^39^ IFN-γ upregulates PD-L1 mRNA and protein in MPM cell lines in vitro,^49^ a finding supported by Khanna *et al*.^48^ Pretreatment with IFN-γ of AB1, a sarcomatoid murine MPM cell line, can sensitize mice to anti-PD-1 plus anti-CTLA-4 therapy, which suggests it may have clinical efficacy when administered alongside ICB.^50^

Overall, MPM-associated CTLs commonly exhibit widespread exhaustion with variable infiltration, but antitumor activity and signaling can be restored in vitro and in vivo. IFN-γ supplementation and TIM-3 inhibition are intriguing therapeutic strategies to invigorate CTL-mediated tumor rejection, supported by initial evidence from preclinical MPM models.

## TAMs in MPM

**Figure 3:**
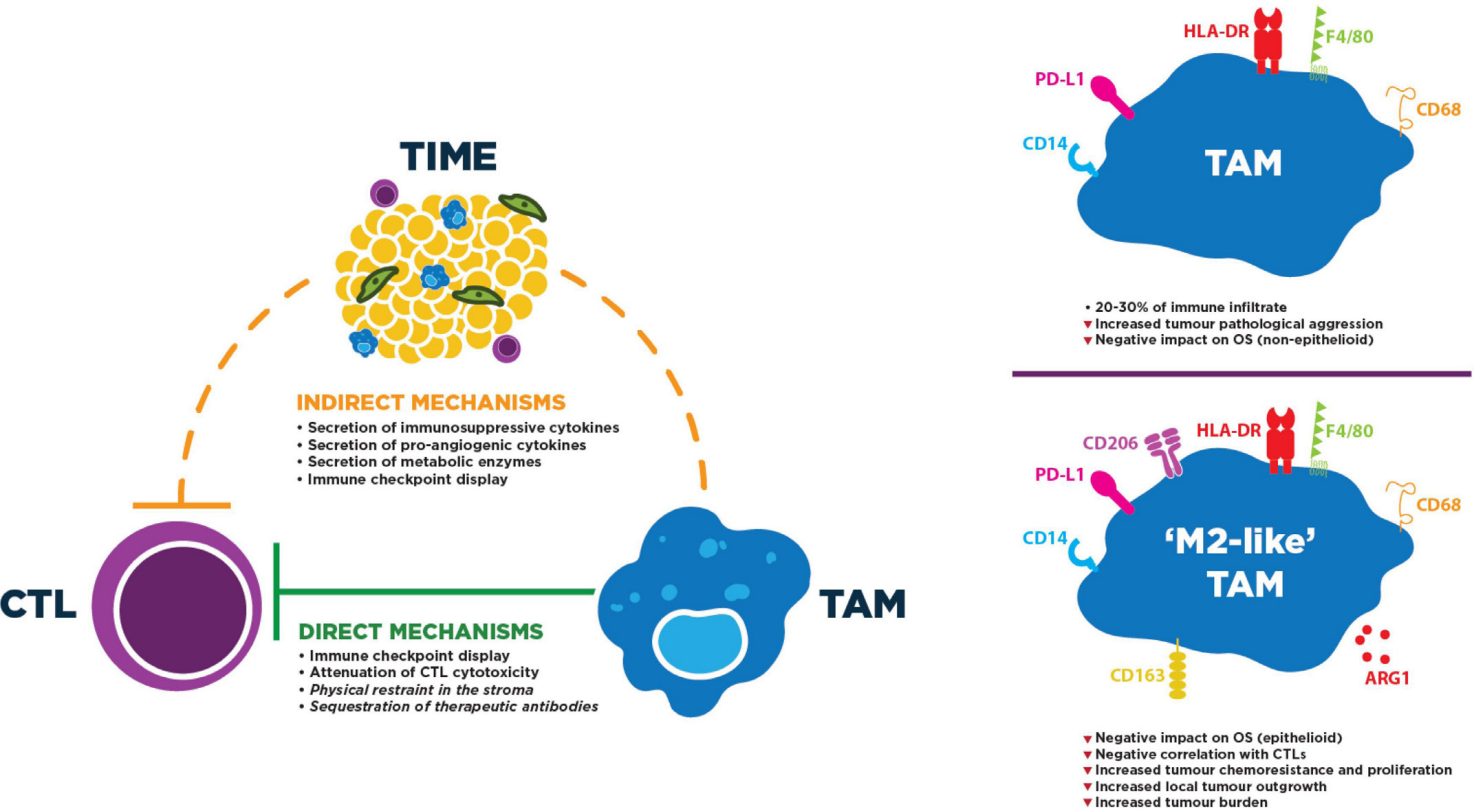
Profile and mechanisms of TAM immunosuppressive activity to directly and/or indirectly repress CTL function. Left: these activities can entail direct contact with CTLs or indirectly induce CTL dysfunction by altering the tumor microenvironment. Mechanisms in italics likely occur, but have not yet been observed, in MPM. Right: M2-like TAMs typically express higher levels of tolerogenic proteins. Although CD163, CD206 and Arg1 have been targeted to identify M2-like TAMs in MPM, markers of TAM classification continue to be controversial. F4/80 is exclusive to mouse TAMs. CTL, cytotoxic T lymphocyte; MPM, malignant pleural mesothelioma; OS, overall survival; TAM, tumor-associated macrophage; TIME, tumor immune microenvironment.

TAMs are the predominant leukocyte population in MPM tumors and overwhelmingly exhibit a protumor phenotype, both fostering tumor growth and mediating CTL repression (figure 3, left panel). They themselves often express ICs, especially PD-L1.^51^ Although immunohistochemistry (IHC)-based research has found comparatively low PD-L1 positivity on tumor-infiltrating leukocytes outside of the trial context,^52^ a recent flow cytometric study revealed that approximately 95% of TAMs (defined as CD14^+^/HLA-DR^high^) were PD-L1^+^, and that positivity was more common in TAMs (and other myeloid-lineage cells) than in non-immune (CD45^−^) cells.^20^ Expression of other ICs on TAMs is still poorly understood in MPM. Meanwhile, TAMs also secrete cytokines such as interleukin (IL)-10 and transforming growth factor beta (TGF-β) to supplement the immunosuppressive milieu surrounding tumors, both of which have been measured at elevated levels in MPM-associated macrophages: the latter also contributes to tumor growth and angiogenesis.^53 54^ Similarly, secretion of arginase and indoleamine 2,3-dioyxgenase act to metabolically starve CTLs and inhibit their cytotoxicity.

More direct mechanisms of TAM-mediated CTL repression have been identified recently; namely, the ability of macrophages to directly impede CTL migration into the tumor nest has been demonstrated. In human lung tumors, stromal CTLs formed lasting interactions with CD11c^+^/CD206^+^ TAMs: lifting these interactions increased CTL motility and tumor-area infiltration.^55^ Similarly, coincubation of tumor-specific CTLs with M2 TAMs polarized in vitro attenuated their cytolytic capabilities against the Meso 34 MPM cell line.^56^ Furthermore, in vivo imaging of immunocompetent mice revealed the disturbing tendency of TAMs to sequester ICB antibodies from the surface of CTLs shortly after their engagement.^57^ Although their activity has not been explored in the trial context, TAMs are recruited by MPM tumors to exclude and repress CTLs, cooling down the TIME to altered phenotypes. Thus, they represent a rational target for novel immunotherapies.

### Characterization and clinical impact of TAMs in MPM

TAMs are often dichotomized into M1 (antitumor) and M2 (protumorigenic and immunosuppressive),^58^ but macrophages exhibit remarkable, microenvironment-dependent plasticity not captured by this dichotomisation.^59^ Consequently, unlike CTLs, discriminating TAM populations of interest is challenging: for instance, one examination of the AE17 murine MPM model identified a TAM population positive for both the immunosuppressive IL-10 and protumoricidal tumour necrosis factor alpha (TNF-α) cytokines.^53^ Differences in human and murine immunology compound this difficulty.^60^ However, the M1/M2 dichotomisation is commonly used, so it will be repeated here to summarize existing studies.

Given their innate plasticity, TAMs are easily persuaded to perform M2-like functions by the predominantly immunosuppressive milieu of the TIME (figure 3, right panel). Indeed, while TAMs account for approximately 20%–30% of the total immune infiltrate,^20 61^ the majority of these are considered immunosuppressive. Some investigations suggest that M2-like TAMs, typically defined as CD163^+^ cells in IHC, are negatively prognostic: for example, a high ratio of infiltrating CD163:CD8 indicated poorer OS in epithelioid MPM,^29^ while an M2-like skewing of phenotype was associated with lower OS in a separate epithelioid cohort.^62^ CD163-expressing TAMs have also been collected from pleural effusions, where they negatively correlated with CTL quantity, though inferences regarding causality cannot be made.^63^ In a separate study, effusion-derived, M2-like TAMs (CD163^high^/CD14^high^) also conferred chemotherapy resistance and improved proliferation on patient-matched cancer cells.^64^ An increased CD163:CD68 ratio has also been associated with more probable local tumor outgrowth,^65^ while in the murine peritoneal mesothelioma model 40L, depletion of M2-like TAMs (F4/80^+^/Arg1^+^) significantly reduced tumor burden.^54^ Furthermore, some studies have discovered that the TAM population imparts poor clinical outcomes irrespective of finer classification: infiltration of cells expressing the accepted pan-TAM marker CD68 has been associated with aggressive pathological features^25^ and poorer OS in non-epithelioid tumors.^61^ This finding is not universal, perhaps due to MPM’s familiar heterogeneity or TAM phenotypical plasticity. Indeed, flow cytometry on CD14^+^ PBMCs forcibly differentiated into macrophages indicated that CD68 and CD163 protein levels were equivalent in dedicated M1 and M2 cells following polarisation in vitro.^66^ Nonetheless, determining whether all TAMs worsen OS, or only an M2-like subgroup, will be vital to maximize effectiveness of future anti-TAM therapeutic strategies.

### Targeting TAMs in novel MPM immunotherapies

Two broad concepts exist in TAM-targeting strategies: reprogramming and depletion. Within depleting strategies, multiple studies in MPM converge on CSF-1R inhibition. Ligation of CSF-1R by CSF-1 facilitates mitogen activated protein kinase (MAPK) signaling within monocytes and ultimately induces differentiation into macrophages.^67^ Notably, there are multiple reports that secretion of CCL2, the primary monocyte-recruiting chemokine, is elevated in both pleural effusions and the circulation of patients with MPM.^64 68 69^ These observations, combined with the protumorigenic and immunoregulatory functions of TAMs, suggest that MPM tumors prioritize continuous recruitment of monocytes to coerce their differentiation into TAMs, and hence, they can secure a consistently procancer TIME to ensure their own survival. Consistent with this hypothesis, CSF-1R inhibition results in various antitumor outcomes. In AB1 (sarcomatoid) and AC29 (epithelioid) murine models, TAM and non-classical (Ly6C^low^) monocyte quantity and incidence of angiogenesis were reduced after CSF-1R inhibition, also allowing increased survival and lower CTL exhaustion incidence when combined with a dendritic cell vaccine.^70^ Similarly, breast tumor-bearing mice experienced higher CTL infiltration and lower tumor burden after treatment with a CSF-1R inhibitor alongside PD-1 blockade.^55^ CSF-1R inhibition also alleviated the repressive effect of M2-polarized TAMs on CTLs to allow MPM cell line killing in vitro.^56^ Alternative depleting agents have also been successfully investigated in MPM models,^54 71^ while our own work has also identified the fibroblast-derived secreted protein STC1 as an additional regulator of TAM differentiation in lung adenocarcinoma.^72^

By contrast, reprogramming strategies attempt to stimulate TAMs to repress their protumor functions and switch to a phagocytic role. A reprogramming function of CSF-1R inhibition has been proposed in glioma, which results in downregulation of M2-like markers,^73^ possibly by diverting classical monocyte maturation from the CSF-1 to the CSF-2 pathway. Although CSF-2 (GM-CSF, granulocyte-macrophage colony-stimulating factor) is currently underexplored in MPM, initial evidence suggests that its abundance may be too low to facilitate this reprogramming function.^70^ Many other distinct agents have been tested for this purpose in murine models of MPM and beyond.^2 53 74–76^ Interestingly, the targeting of galectin-9 induced TAM reprogramming and direct MPM cell apoptosis: galectin-9 is also a TIM-3 ligand, suggesting that this could represent a multifaceted therapeutic avenue.^77^ In summary, TAM targeting has been explored preclinically in MPM, and depletion via CSF-1R inhibition, the subject of later-phase trials in other malignancies,^78^ may be productive in MPM. Their predominance in the TIME attests to their importance to MPM tumors, while their powerful immunosuppressive activities suggest that ICB effectiveness may be enhanced by TAM inhibition or repolarisation, as demonstrated preclinically.

## Myeloid-derived suppressor cells

Despite efforts to characterize MDSCs reproducibly,^79^ their biology is incompletely understood. The current MDSC paradigm describes two immature, immunosuppressive myeloid subpopulations: the predominant polymorphonuclear myeloid-derived suppressor cells (PMN-MDSCs) and less common monocytic myeloid-derived suppressor cells (M-MDSCs) types, classified according to lineage-specific biomarker expression.^80^

Chemotherapies can deplete circulating and MPM-infiltrating MDSCs to lift their protumorigenic effect. In one murine study, Gr1^+^ MDSC-like infiltration of AB1-HA tumors was significantly reduced by cisplatin-pemetrexed, and Gr1^+^ depletion alongside PD-1 blockade resulted in slower tumor growth.^81^ Systemic depletion of MDSCs has also been noted.^53 82^ In humans, gemcitabine treatment can depress CD11b^+^/CD33^+^/HLA-DR^−^ MDSC numbers in PBMCs, associated with increased T-cell proliferation.^83^

PMN-MDSCs can directly inhibit infiltrating T-cell proliferation and IFN-γ secretion, mediated by MPM-derived GM-CSF (CSF-2) and MDSC-derived reactive oxygen species.^84^ Inhibition of either of these restores the T-cell effector phenotype.^84^ Correlation of MDSC infiltration with CTL exhaustion has been shown, hinting at a possible suppressive mechanism,^85^ while effector CTLs can retaliate to directly kill MDSCs. Crucially, both PMN-MDSCs and M-MDSCs have been associated with worse OS and progression-free survival in MPM^30^ and merit further exploration as therapeutic targets. Like TAMs, preclinical studies suggest that MDSC depletion may synergize with existing ICB therapy.

## Regulatory T lymphocytes

T_regs_ are a relatively well-understood immunosuppressive population in MPM, typically identified by the specific transcription factor FOXP3. In healthy tissues, T_regs_ mediate immune tolerance, especially through IL-10 and TGF-β secretion, to avert autoreactive responses. When displaying a CD4^+^/FOXP3^+^/CD25^hi^/CD127^low^ phenotype, T_regs_ are generally considered to be committed to their immunosuppressive function, while CD25^lo^ T_regs_ can suppress their FOXP3 activity and convert to helper T cells under proinflammatory conditions.^86^

Like other T cells, T_regs_ also express CTLA-4: notably, T_reg_ CTLA-4 positivity of approximately 70% has been reported for MPM, which is substantially greater than that found on MPM-associated CTLs^20^ and may provide further mechanistic insight into the clinical results of CTLA-4 monotherapy in MPM. Additionally, the same report identified T_regs_ cells as being associated with depressed IFN-γ production in CTLs,^20^ in agreement with earlier murine studies,^87 88^ indicating subdued CTL activation that may translate to impaired tumor killing. Consistent with this, large, intratumoral T_reg_ populations are frequently associated with reduced patient OS, even in multivariate analysis,^30 31 89^ though the clinical impact of peripheral T_regs_ is less clear. Besides awakening CTL activity, T_reg_ depletion commonly results in tumor growth inhibition in MPM mouse models^88 90^ and has been associated with an influx of CTLs and memory T cells to the TIME.^91^ Similarly, a report describing ICB applied to antigen-specific T cells acknowledged a smaller T_reg_ population, and concomitant higher CTL population, in responders compared with refractive AB1-HA tumors.^92^ MPM-associated T_regs_ therefore perform both suppressive and exclusionary functions, much like TAMs, but may not be sufficient to render tumors cold when acting alone.^93^

Despite considerable evidence of their immunosuppressive impact, anti-T_reg_ strategies for MPM have stagnated in the preclinical phase. Reports suggest that T_reg_ gene expression signatures are more prevalent in neoantigen-rich tumors accompanied by low-diversity T-cell receptor repertoires^94^ and in those enriched for clonal neoantigens.^26^ Hence, T_regs_ may be preferentially recruited to clonally immunogenic MPMs and would thus represent an attractive therapeutic target alongside ICB in at least this subset of patients.

## MPM-intrinsic modulation of the TIME

### Programmed death - ligand 1 (PD-L1)

As the primary ligand for PD-1, PD-L1 expression represents a direct immunosuppressive mechanism for tumors. PD-L1 has become established as a predictive biomarker PD-1 blockade in lung cancer. The evidence in MPM had been at best equivocal, but recent phase III trials demonstrate that PD-L1 cannot be considered predictive in MPM at present. Tumor PD-L1 positivity varies between 20% and 80% overall in MPM, averaging approximately 40%, while evidence of its prognostic impact is mixed. PD-L1 expression of ≥1% was associated with inferior OS in the chemotherapy arm of CheckMate 743,^95^ indicating that it was negatively prognostic for this treatment. A meta-analysis of 11 IHC studies reported PD-L1 as a negative prognostic factor (HR=1.50).^96^ However, this meta-analysis did not take into account the higher rate of PD-L1 expression in non-epithelioid MPM, which carries a poorer prognosis.^52 96–100^ Accordingly, an investigation within the MAPS trial concluded that PD-L1’s prognostic power was lost when accounting for covariates such as histology.^101^ Therefore, alternative biomarkers will likely be required to stratify patients with ICB in MPM.

### Neoantigen burden and diversity

Antitumor CTLs depend on neoantigen display by tumors to identify their cellular targets. Elevated tumor mutation burden (TMB) is thought to directly increase the frequency of immunogenic neoantigen display in probabilistic fashion, resulting in increased tumor-specific killing once unleashed by ICB. A high TMB is an established predictor of response to PD-1 axis ICB in melanoma and non-small cell lung cancer.^14 102 103^ Initially, mesothelioma was generally considered a cancer of very low TMB according to whole exome sequencing,^104–106^ but leveraging alternative techniques such as mate-pair sequencing and comparative genomic hybridisation have uncovered greater rates of genomic alteration, which is expected of a malignancy demonstrating modest responses to ICB^107–109^ and may prove predictive.

Several studies have identified putative neoantigens to explain clinical responses in MPM, with chromothripsis as a potential mechanism.^94 108^ One further related a predicted neoantigen to robust antitumor immunity.^110^ Nonetheless, CTLs also rely on their continued display in the MHC I context to induce apoptosis. MHC genes *HLA-A*, *HLA-B* and *HLA-C* are especially susceptible to alteration, with even human leukocyte antigen loss of heterozygosity (HLA-LOH) impairing CTL-mediated immunosurveillance in the TracerX cohort.^111^ We recently identified HLA-LOH in 23% of patients with MPM undergoing surgery, all of which were late evolutionary events.^26^ Moreover, they also displayed a more diverse predicted neoantigen repertoire than HLA-intact tumors, indicating higher susceptibility to tumor-specific CTLs. Hence, these HLA-LOH events may constitute an acquired immune escape mechanism in MPM that merits examination in ICB-resistant tumors.

### Is MPM a cold cancer?

Tumor-intrinsic factors such as mutation and neoantigen burden are typically considered the primary drivers of CTL infiltration and activity, which indicate effective antitumor immunity. Due to its relatively modest ICB response rates, MPM might be considered a ‘cold’ cancer. However, the first steps in characterizing its TIME suggest otherwise.

‘Hot’ tumors are defined as those with a sizeable CTL infiltrate not only into the TIME,^112^ but also deeper beyond the invasive margin, while cold tumors lack both and represent widespread immunological ignorance, driven by defects in activation and recognition of neoantigens.^113 114^ Hot tumors typically also feature substantial pre-existing cytolytic activity and high PD-L1 expression to evade it^115 116^: deeper leukocyte infiltration towards PD-L1^+^ MPM cells has been demonstrated using multiplex, fluorescent IHC.^117^ In MPM, the temperature of tumors may also relate to their evolutionary trajectory, with colder tumors described by simpler, linear genomic phylogenies.^26^

**Figure 4:**
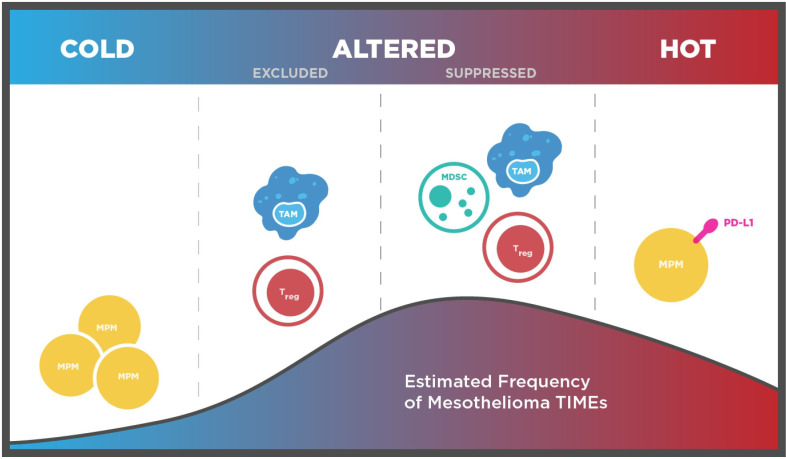
Intermediate inflammatory states define the majority of MPM TIMEs. Area under the curve denotes estimated frequency of TIME phenotypes. Quantitative and spatial immunophenotyping data suggest most MPM TIMEs bear the ‘altered’ phenotype, while hot tumors are uncommon and cold tumors are rare. TAMs, particularly M2-like TAMs, T_regs_ and MDSCs suppress infiltrated tumors, while TAMs and T_regs_ can actively exclude cytotoxic T lymphocytes. MDSC, myeloid-derived suppressor cell; MPM, malignant pleural mesothelioma; TAM, tumor-associated macrophage; TIME, tumor immune microenvironment; T_reg_, regulatory T lymphocyte.

Between these extremes, tumors are considered ‘altered’, further categorized as ‘immunosuppressed’ (with CTLs infiltrating into tumor nests, but without meaningful activity due to factors described later) or ‘excluded’ (featuring infiltration into the TIME, but rarely beyond the invasive margin).^113^ Quantitative and spatial evidence accumulated so far indicates that MPMs are rarely cold, but commonly altered and less frequently hot (figure 4),^26 117^ with PD-L1 a potential hot TIME marker.^21 25^ The immunoregulatory impact of V-domain immunoglobulin suppressor of T-cell activation, which is highly expressed on epithelioid MPM compared with other tumors, remains to be elucidated.^118 119^ In all, MPM TIMEs are rarely cold and therefore may be amenable to therapeutic interventions that enhance their immunogenicity. Hot TIMEs are also expected to bear biomarkers of therapeutic resistance or sensitivity and thus warrant further characterisation.

## Conclusion

MPMs exhibit a heterogeneous TIME, harboring tumor-reactive CTLs balanced by a substantial myeloid component, which is dominated by immunosuppressive and protumorigenic TAMs. CTLs exhibit widespread exhaustion, which could be combated by coinhibition of checkpoint receptors including TIM-3 and TIGIT alongside PD-1. Depleting or reprogramming TAMs may potentiate effector CTL function and facilitate therapeutic tumor suppression, while MDSCs, T_regs_ and tumor-derived factors like CCL2 represent potential therapeutic targets. Finally, strong evidence suggests chemotherapy is immunomodulatory and could enhance ICB by inflaming cool TIMEs.
